# The spider genus *Pterotricha* in Iran, with the description of a new genus (Araneae, Gnaphosidae)

**DOI:** 10.3897/zookeys.777.26745

**Published:** 2018-07-30

**Authors:** Alireza Zamani, Marjan Seiedy, Alireza Saboori, Yuri M. Marusik

**Affiliations:** 1 School of Biology, College of Sciences, University of Tehran, Tehran, Iran; 2 Jalal Afshar Zoological Museum, Department of Plant Protection, Faculty of Agriculture, University of Tehran, Karaj, Iran; 3 Institute for Biological Problems of the North RAS, Portovaya Str. 18, Magadan 685000, Russia; 4 Department of Zoology & Entomology, University of the Free State, Bloemfontein 9300, South Africa

**Keywords:** Aranei, ground spiders, Iranian Plateau, *Iranotricha* gen. n., new species, new synonymy

## Abstract

The spider genus *Pterotricha* Kulczyński, 1903 (Gnaphosidae) is surveyed in Iran. To date, three species of this genus were known in the country: *P.loeffleri* (Roewer, 1955), *P.lentiginosa* (C. L. Koch, 1837) and *P.pseudoparasyriaca* Nuruyeva & Huseynov, 2016. Here two new species are described, *P.kovblyuki* Zamani & Marusik, **sp. n.** (♂, western Iran) and *P.montana* Zamani & Marusik, **sp. n.** (♀, central and southwestern Iran), and P.cf.dalmasi Fage, 1929 (from Algeria to Jordan) is reported in Iran for the first time. *Pterotrichatikaderi* Gajbe, 1983 **syn. n.** (India) and *P.loeffleri* (Roewer, 1955), **syn. n.** are synonymized with *P.strandi* Spassky, 1936 (hitherto known from Turkmenistan only). The record of *P.lentiginosa* from Iran is apparently based on misidentification. A distribution map of the genus in Iran with new provincial records is provided. In addition, *Iranotricha* Zamani & Marusik, **gen. n.**, a new genus closely related to *Pterotricha*, is described from southeastern Iran with the type species *I.lutensis* Zamani & Marusik, **sp. n.**

## Introduction

Gnaphosidae is a large, globally distributed family currently comprising 2196 extant species in 124 genera (WSC 2018). Of these, the Old World genus *Pterotricha* Kulczyński, 1903, currently comprises 39 valid species primarily distributed in arid and semi-arid habitats of the Middle East, North Africa, and central Asia (WSC 2018, Levy 1995). These are medium-sized (5–13 mm) Gnaphosinae spiders, which are distinguished from other genera of the subfamily by their very long, rigid cylindrical, tube-shaped anterior spinnerets extending far beyond the other spinnerets (Levy 1995). The first Iranian record of this genus was provided by Roewer (1955) who described *P.loeffleri* (sub *Bobineusl.*, Cithaeronidae) and recorded *P.lentiginosa* (C. L. Koch, 1837) for the first time from Iran. Later, *P.pseudoparasyriaca* Nuruyeva & Huseynov, 2016 was recorded in the country by Zamani et al. (2017). The goals of this paper are to survey all records of this genus in Iran, including two species new to science and one new to the Iranian fauna, and describe a closely related new genus and its type species.

## Materials and methods

Specimens were photographed using an Olympus Camedia E‐520 camera attached to an Olympus SZX16 stereomicroscope or to the eye‐piece of an Olympus BH‐2 transmission microscope and a SEM JEOL JSM-5200 scanning electron microscope. Digital images were prepared using “CombineZP” image stacking software (http://www.hadleyweb.pwp.blueyonder.co.uk/). Illustrations of internal genitalia were made after clearing in 10% KOH aqueous solution and exposure for a few minutes in an alcohol/water solution of Chlorazol Black. Lengths of leg segments were measured on the dorsal side. Leg measurements are listed as total length (femur, patella, tibia, metatarsus, tarsus). The description of the palp refers to the left one. All measurements are given in millimetres. Abbreviations not explained in the text are listed below:

**ALS** anterior lateral spinneret

**AME** anterior median eye

**ALE** anterior lateral eye

**PME** posterior median eye

**PLE** posterior lateral eye

### Depositories

**AZMI** Agricultural Zoology Museum, Iranian Research Institute of Plant Protection, Tehran, Iran (A. Khalegizadeh)

**EMSUM** Entomological Museum of Shiraz University of Medical Sciences, Shiraz, Iran (A. Soltani)

**MMUE** the Manchester Museum of the University of Manchester, Manchester, UK (D. Logunov)

**SMF**Senckenberg Museum, Frankfurt am Main, Germany (J. Altmann)

**ZMFUM** Zoological Museum of Ferdowsi University of Mashhad, Mashhad, Iran (O. Mirshamsi)

**ZMMU**Zoological Museum of the Moscow State University, Moscow, Russia (K. Mikhailov)

**ZUCT**University of Tehran, Tehran, Iran (A. Sari)

**ZSI** National Zoological Collections, Zoological Survey of India, Calcutta, India

## Taxonomy

### Gnaphosidae Pocock, 1898

#### Pterotricha

Taxon classificationAnimaliaAraneaeGnaphosidae

Kulczyński, 1903: 43.

Bobineus Roewer 1955: 773.
Pterotricha : Dalmas 1921: 248; Levy 1995: 944; Murphy 2007: 122–123.

##### Type species.

*Aranealentiginosa* C. L. Koch, 1837.

##### Comments.

**Figure 1.**
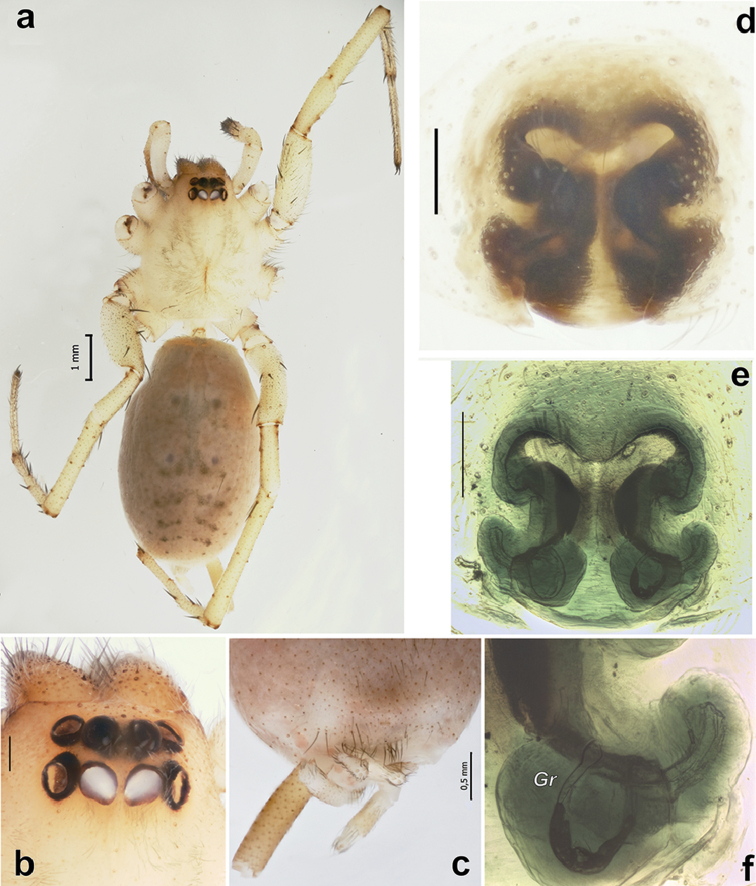
Female of *Pterotrichadalmasi* (?). **a** habitus, dorsal **b** cephalic part of carapace, dorsal **c** spinnerets, ventral **d, e** epigyne, ventral and dorsal **f** right receptacle, dorsal. Scale bars = 0.2 mm if not otherwise indicated. Abbreviation: *Gr* receptacular gland.

The genus belongs to Gnaphosinae, and like all other members of this subfamily has a serrated keel on chelicera. It is easily distinguished from all other Gnaphosinae by long to very long spinnerets (Figs 1c, 4a, 6a–b, 10b–c, 11g), the male palp with squarrose tibial apophysis and a heavily sclerotized (non-membranous) and pointed “conductor”. Most of the species have a stylus on embolus (vs. lacking in all other genera).

##### Distribution.

The genus is known from Spain to South Africa, to western India, with a single, doubtful record of *Pterotrichasaga* (Dönitz & Strand, 1906) from Japan (most likely belonging to *Callilepis* Westring, 1874).

#### Pterotricha cf. dalmasi

Taxon classificationAnimaliaAraneaeGnaphosidae

Fage, 1929

Figs 1 , 16

Pterotricha dalmasi : Levy 1995: 948, f. 71–76 (♂♀).

##### Material examined.

IRAN: 1♀ (ZUCT), *Hormozgan Province*: Minab, sand dunes of Minab beach, February 2018 (A. Zamani).

##### Diagnosis.

This species closely resembles *P.conspersa* (O. Pickard–Cambridge, 1876). Males are diagnosed by the different shape of the base of the tegular apophysis (sub-circular in *P.dalmasi* vs. irregularly shaped in *P.conspersa*) and the retrolateral tibial apophysis (more massive in *P.conspersa*), while females can be distinguished by the different shape of the epigynal septum (anterior depression pointed posteriorly and median curves pointed anteriorly or anterolaterally in *P.dalmasi* (Figs 1d, e) vs. the anterior depression pointed anteriorly and median curves pointed posteriorly in *P.conspersa*) (Levy 1995).

##### Description.

Well described by Levy (1995).

##### Comments.

The species was described from males. Levy (1995) was the first to describe females of this species. He provided figures of two “forms” of the epigyne (Levy 1995: figs 74–75). The epigyne illustrated on fig. 75 is rather similar to the epigyne of our specimen. Apparently, the small (tiny) receptacular gland present in the Iranian specimen was overlooked in the specimens from Israel, and perhaps the female specimens illustrated by Levy (1995) belong to two different species. A lack of samples containing both males and females does not allow us to conclude which of the two “forms” of the females are conspecific with *P.dalmasi*.

##### Records in Iran.

Hormozgan (Fig. 16).

##### Distribution.

From Algeria to Iran (first record), south to Sudan and Saudi Arabia (WSC 2018).

#### Pterotricha kovblyuki

Taxon classificationAnimaliaAraneaeGnaphosidae

Zamani & Marusik sp. n.

http://zoobank.org/E55B2388-5DB7-4BAE-8355-5F3AB9072CD3

Figs 2 , 16

##### Type.

Holotype ♂ (AZMI), IRAN: *Ilam Province*: Mehran County, 2001 (F. Mozaffarian).

##### Etymology.

This species is named after the Ukrainian arachnologist Mykola Kovblyuk in recognition of his contributions to the taxonomy of gnaphosid spiders.

##### Diagnosis.

**Figure 2.**
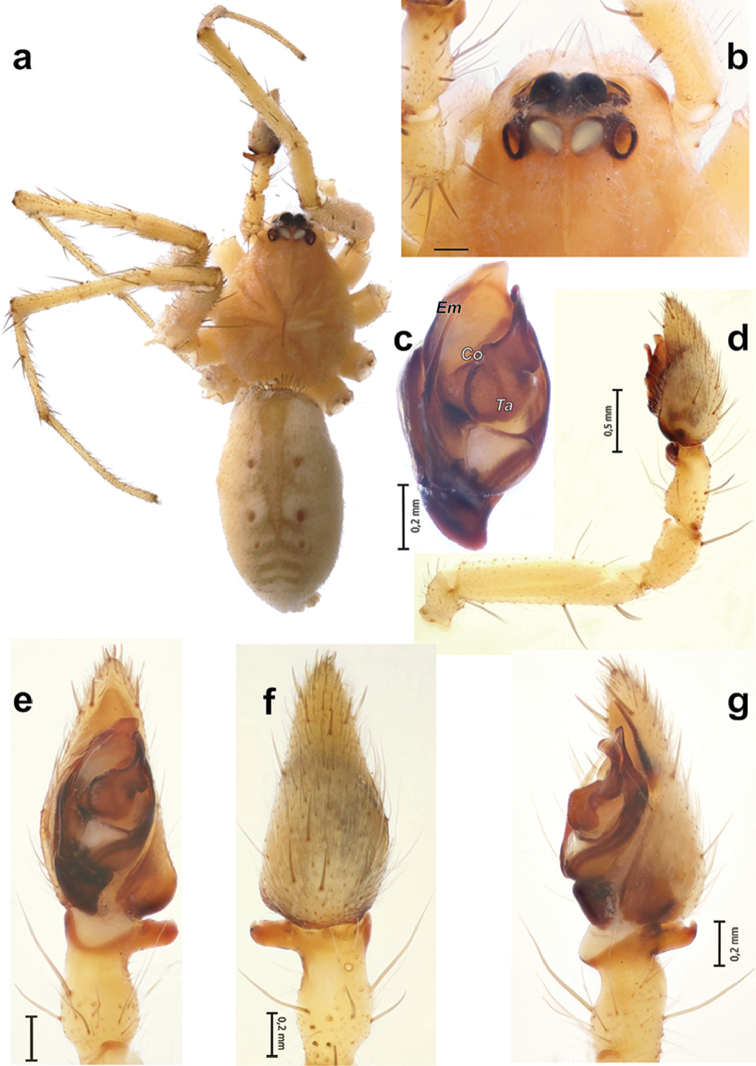
*Pterotrichakovblyuki* Zamani & Marusik sp. n., male. **a** habitus, dorsal **b** cephalic part of carapace, dorsal; **c** bulb, ventrolateral **d** palp, retrolateral **e–g** palp, ventral, dorsal and retrolateral. Scale bars = 0.2 mm if not otherwise indicated. Abbreviations: *Co* conductor; *Em* embolus; *Ta* tegular apophysis.

The new species is most similar to *P.dalmasi* by lacking a stylus on the embolus, the similar shape of the tegular apophysis and the tibial apophysis with a spine like tip (cf. Figs 2e–g and figs 71–73 in Levy 1995). The two species can be separated by the thinner tip of the conductor and the tegular apophysis which is longer than wide in the new species, vs. wider than long in *P.dalmasi* (cf. Fig. 2e and figs 71–73 in Levy 1995).

**Description.** Male. Total length 6.7. Carapace 2.8 long, 2.25 wide. Eye sizes and interdistances: AME: 0.21, ALE: 0.17, PME: 0.24, PLE: 0.20, PME–PME: 0.05. Carapace, sternum, labium, chelicerae, and maxillae light brown without any distinct patterns, with darkening in the ocular area. Abdomen light grey with distinct pale cardiac mark with three pairs of dots on either side. Legs the same colour as the carapace and without annulations. Scopula on metatarsi and tarsi indistinct. Tarsi of legs I-II (legs III and IV missing) with cuticular cracks (pseudosegmented). Leg measurements: I: 12.1 (3.1, 1.25, 2.5, 3.2, 2.05), II: 12.55 (2.95, 1.2, 2.35, 3.65, 2.4), III: absent, IV: absent.

Palp as in Figs 2c–g; patella almost as long as tibia, patella+tibia as long as cymbium; tibial apophysis with one arm, posterior part rounded, anterior part with a spine like tip; tegular apophysis (*Ta*) longer than wide with retrolateral lobe and large base; conductor (*Co*) large, tip rounded; embolus (*Em*) simple and without a stylus.

Female. Unknown.

##### Record in Iran.

Ilam (Figure 16).

##### Distribution.

Western Iran.

#### Pterotricha lentiginosa

Taxon classificationAnimaliaAraneaeGnaphosidae

(C. L. Koch, 1837)

Pterotricha lentiginosa : Roewer 1955: 772.

##### Comments.

This species was recorded in Iran (from two localities in the provinces of East Azarbayjan and Fars) by Roewer (1955) based on two female specimens. Considering Roewer’s well-known taxonomic mistakes, and that the Iranian record is considerably far from its known range (Crete is the easternmost known locality), it is probable that this species was misidentified (Mozaffarian and Marusik 2001). Unfortunately, the specimens were not located at SMF and are probably lost (Julia Altmann pers. comm.).

#### Pterotricha montana

Taxon classificationAnimaliaAraneaeGnaphosidae

Zamani & Marusik sp. n.

http://zoobank.org/4C946927-3277-4247-8FAE-867F36E799BD

Figs 3 , 11g , 16

##### Types.

Holotype ♀ (MMUE), IRAN: *Kohgiluyeh & Boyer Ahmad Province*: Semoron, May 2017 (A. Hosseinpour); Paratypes: ♀ (EMSUM), IRAN: *Kohgiluyeh & Boyer Ahmad Province*: Shadegan, May 2017 (A. Hosseinpour); ♀ (ZUCT), IRAN: *Isfahan Province*: Qamsar & Barzok Protected Area, 55 km SW of Qamsar, 14 km NE Kamoo, Gargash Mountain, 33°39'59"N, 51°19'44"E, 3302 m, May 2016 (P. Ponel).

##### Etymology.

The specific epithet refers to the montane habitat of the species.

##### Diagnosis.

**Figure 3.**
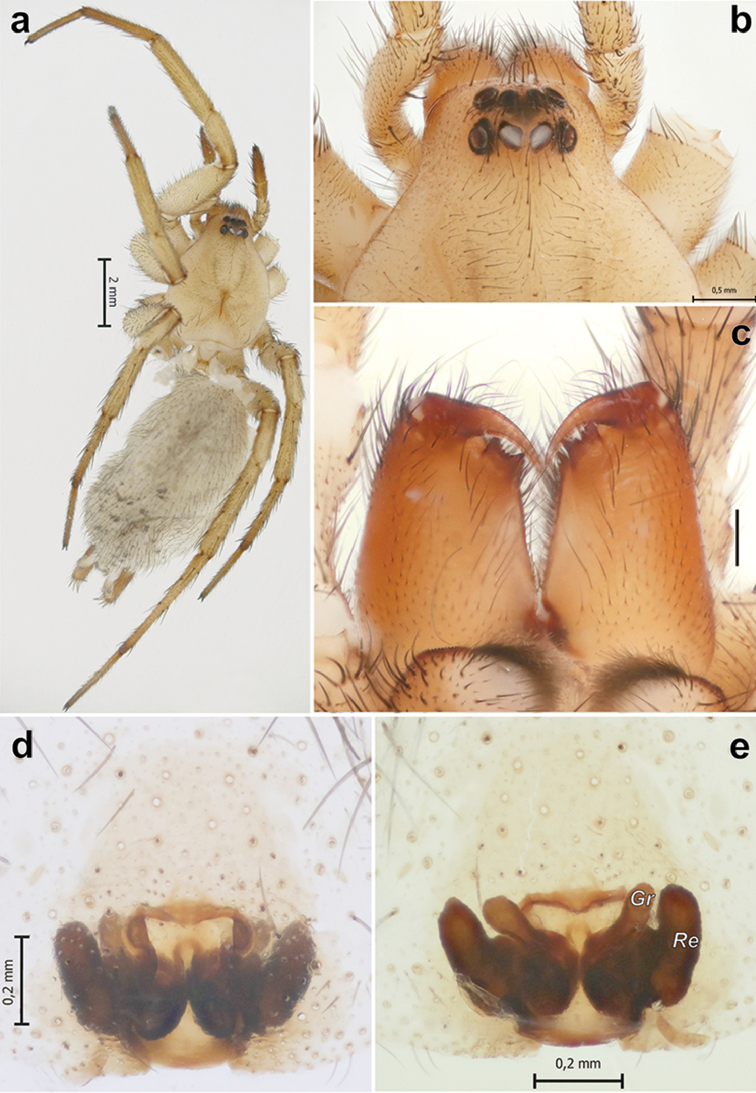
*Pterotrichamontana* Zamani & Marusik sp. n., female. **a** habitus, dorsal **b** anterior part of prosoma, dorsal **c** chelicerae, retrolateral **d, e** epigyne, ventral and dorsal. Scale bar = 0.2 mm if not otherwise indicated. Abbreviations: *Gr* receptacular gland; *Re* receptacle.

This species differs from other congeners by the epigynal plate being wider than long (vs. longer than wide in the remaining species) and the short septum (as long as receptacle length and as long as wide vs. a long septum that is longer than wide and longer than receptacle) (Figs 3d, e).

##### Description.

Female (holotype). Total length 10.95. Carapace 4.1 long, 1.65 wide. Eye sizes and interdistances: AME: 0.14, ALE: 0.20, PME: 0.21, PLE: 0.17, PME-PME: 0.05. Carapace, sternum, labium, chelicerae, and maxillae light brown without any distinct patterns, with scattered short setae and darkening in the ocular area. Chelicerae with one anterior tooth and bifurcated posterior keel. Abdomen light grey with short grey setae and scattered dark patches dorsally. Anterior lateral spinnerets dark brown, relatively short, 3.7 x longer than wide, spaced by less than two diameters of a single ALS, with long spigots of the piriform glands. Legs yellow. Leg measurements: I: 12.85 (3.55, 1.7, 2.7, 2.7, 2.2), II: 12 (3.2, 1.50, 2.55, 2.6, 2.15), III: 11.4 (3.05, 1.45, 2.25, 3.0, 1.65), IV: 14.9 (3.9, 1.55, 3.15, 4.1, 2.2).

Epigyne as in Figs 3d–e; sclerotized part wider than long, septum short, about the length of the receptacle, as long as wide; fovea square; receptacles (*Re*) elongate oval, diverging anteriorly, receptacular gland (*Gr*) massive.

Male. Unknown.

##### Ecology.

This species lives in the mountainous plains of Zagros Mountains.

##### Records in Iran.

Isfahan, Kohgiluyeh & Boyer-Ahmad (Figure 16).

##### Distribution.

Central and southwestern Iran.

#### Pterotricha pseudoparasyriaca

Taxon classificationAnimaliaAraneaeGnaphosidae

Nuruyeva & Huseynov, 2016

Figs 4 , 5 , 16

Pterotricha pseudoparasyriaca Nuruyeva & Huseynov, 2016: 214, f. 1–5, 11–15 (♂♀); Zamani et al. 2017: 63, f. 3B (♂).

##### Material examined.

IRAN: 1♀ (ZMFUM), *Ardebil Province*: Aghkand-Khalkhak Rd., Karoo Vil., 37°24'3.6"N 48°07'4.8"E, 30 May 2017 (A. Abedini); 1♂ 1♀ (ZUCT), *Kordestan Province*: Sanandaj, Noshur, May 2017 (A. Zamani); 2♀ (ZUCT), *Kordestan Province*: Marivan, Bardeh Bakakar, May 2017 (A. Zamani).

##### Diagnosis.

**Figure 4.**
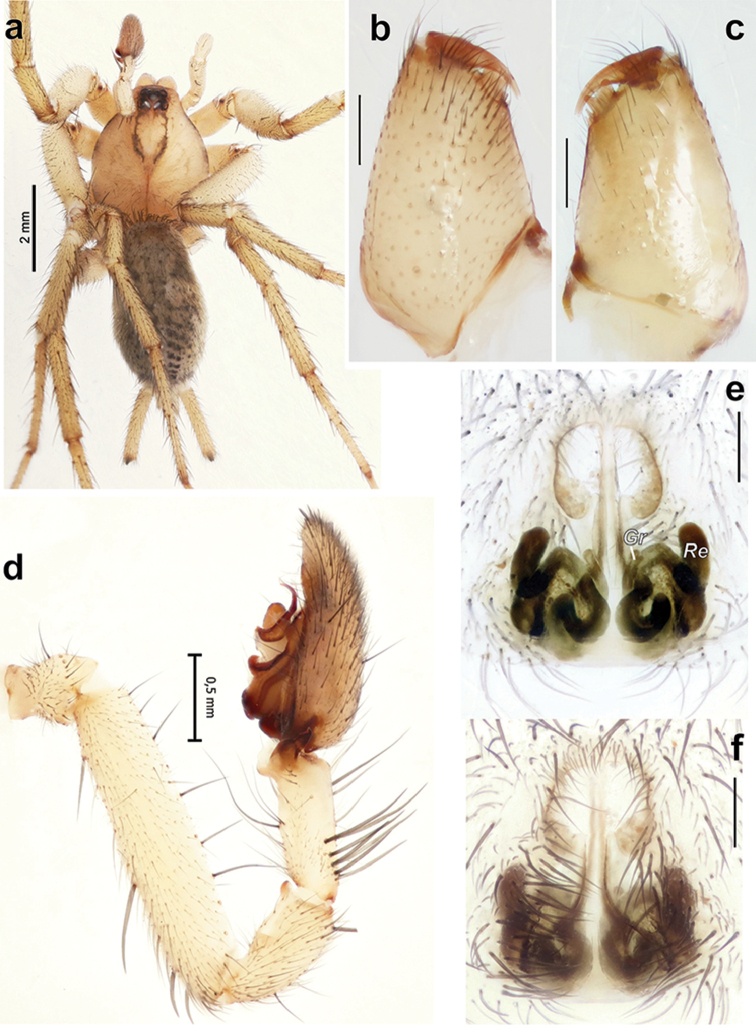
*Pterotrichapseudoparasyriaca*. **a** male habitus, dorsal **b–c** male chelicera, pro- and retrolateral **d** palp, retrolateral **e, f** epigyne, dorsal and ventral. Scale bars 0.2 mm if not otherwise indicated. Abbreviations: *Gr* receptacular gland; *Re* receptacle.

**Figure 5.**
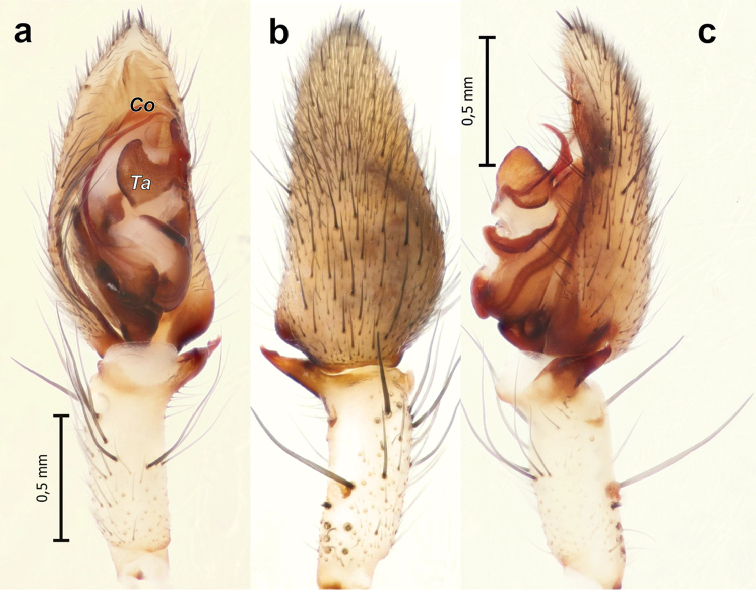
Male palp of *Pterotrichapseudoparasyriaca*. **a** ventral **b** dorsal **c** retrolateral. Abbreviations: *Co* conductor; *Ta* tegular apophysis.

Among Iranian *Pterotricha*, this species is most similar to *P.strandi*, which has a stylus of the embolus, a broad conductor, and a long septum. Males of the two species differ by the shape of the tibial apophysis (tapering in *P.pseudoparasyriaca* vs. rectangular in *P.strandi*), a blunt tip of conductor in *P.pseudoparasyriaca* vs. a sharply pointed tip in *P.strandi*, as well as by the shape of the tegular apophysis (with an elongate base in *P.pseudoparasyriaca* vs. unmodified in *P.strandi*) (Figs 4d, 5a–c). Females of *P.pseudoparasyriaca* are easily distinguished from those of *P.strandi* by having a looped copulatory duct and a distinct, square epigynal fovea vs. copulatory duct short, unlooped and indistinct fovea (Figure 4e, f).

##### Description.

Well-described by Nuruyeva and Huseynov (2016).

##### Records in Iran.

Zanjan. New records: Ardebil, Kordestan (Figure 16).

##### Distribution.

Central eastern and southeastern Azerbaijan, western and northwestern Iran.

#### Pterotricha strandi

Taxon classificationAnimaliaAraneaeGnaphosidae

Spassky, 1936

**Figure 6.**
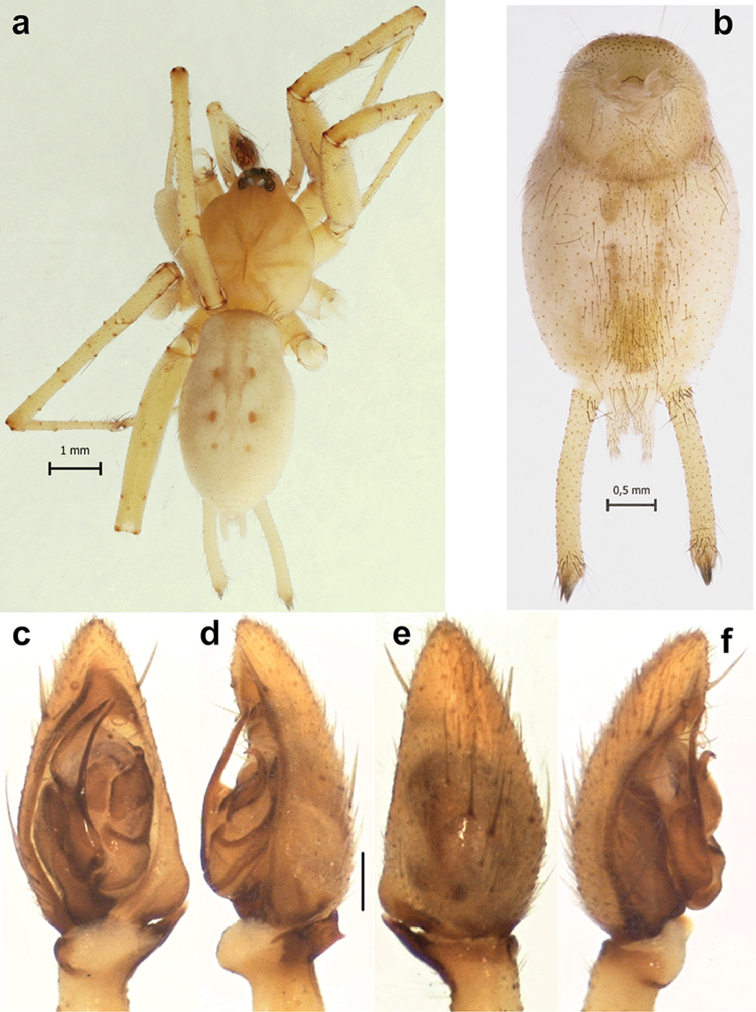
Habitus of male *Pterotrichastrandi* (**a–b**) and male palp of the holotype of *P.tikaderi* (**c–f**). **a** whole specimen, dorsal **b** abdomen, ventral **c–f** ventral, retrolateral, dorsal and prolateral. Scale bar = 0.2 mm if not otherwise indicated.

**Figure 7.**
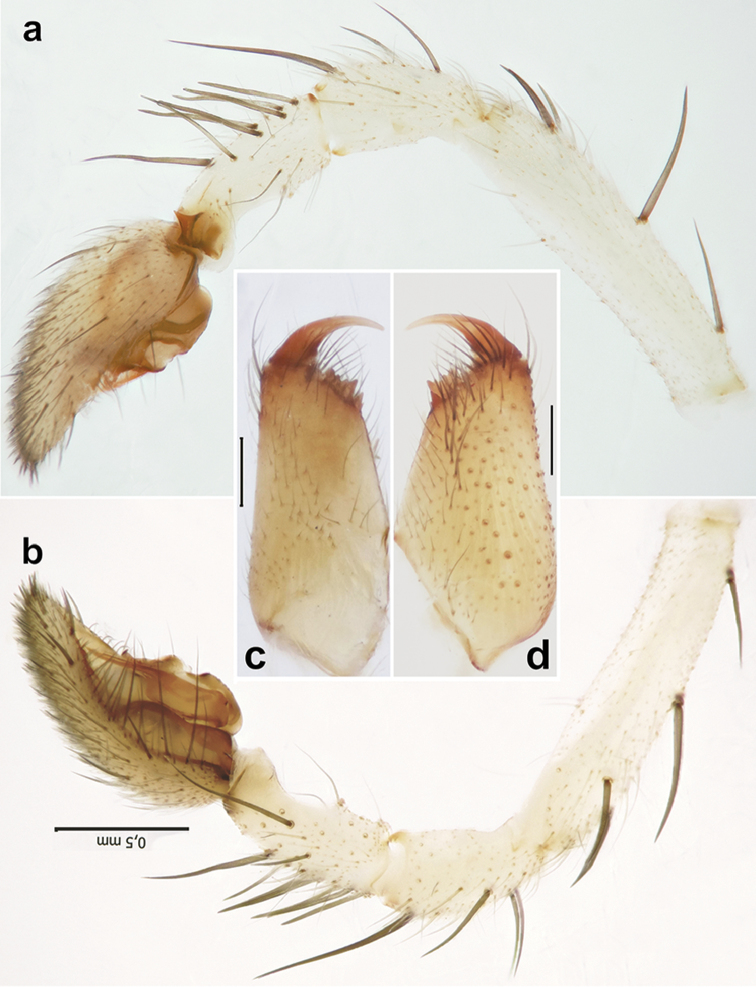
Male of *Pterotrichastrandi*. **a, b** palp, retro- and prolateral **c, d** chelicera, retro- and prolateral. Scale bars = 0.2 mm if not otherwise indicated.

**Figure 8.**
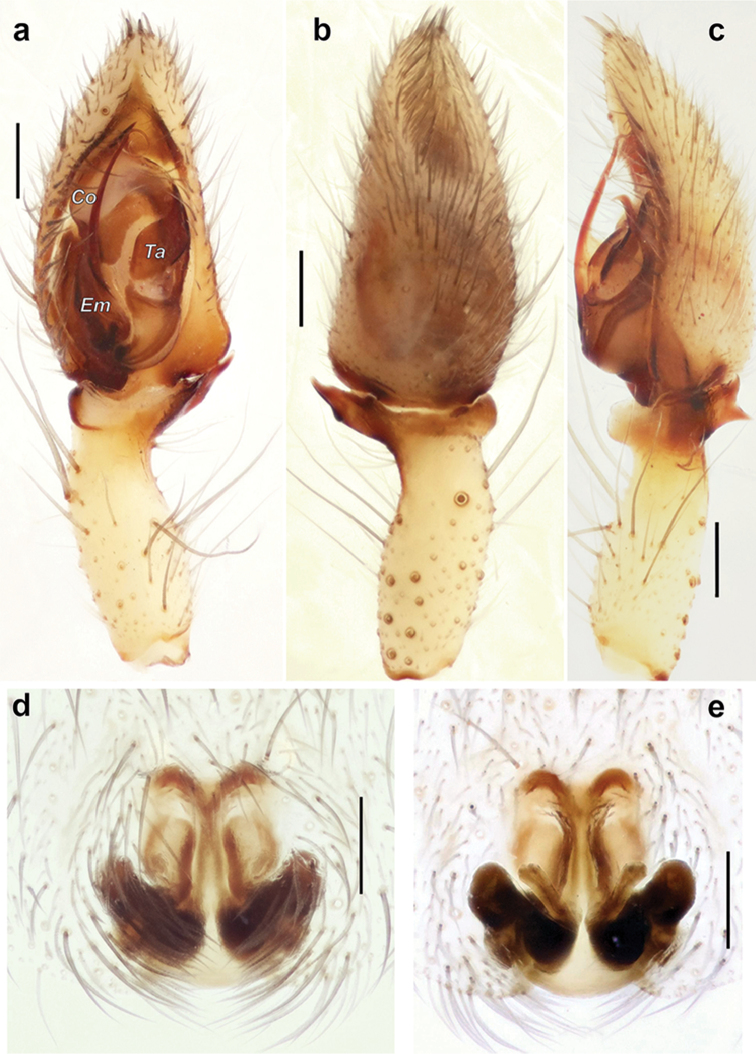
Copulatory organs of *Pterotrichastrandi*. **a–c** male palp, ventral, dorsal and retrolateral **d, e** epigyne, ventral and dorsal. Scale bars = 0.2 mm. Abbreviations: *Co* conductor; *Em* embolus; *Ta* tegular apophysis.

**Figure 9.**
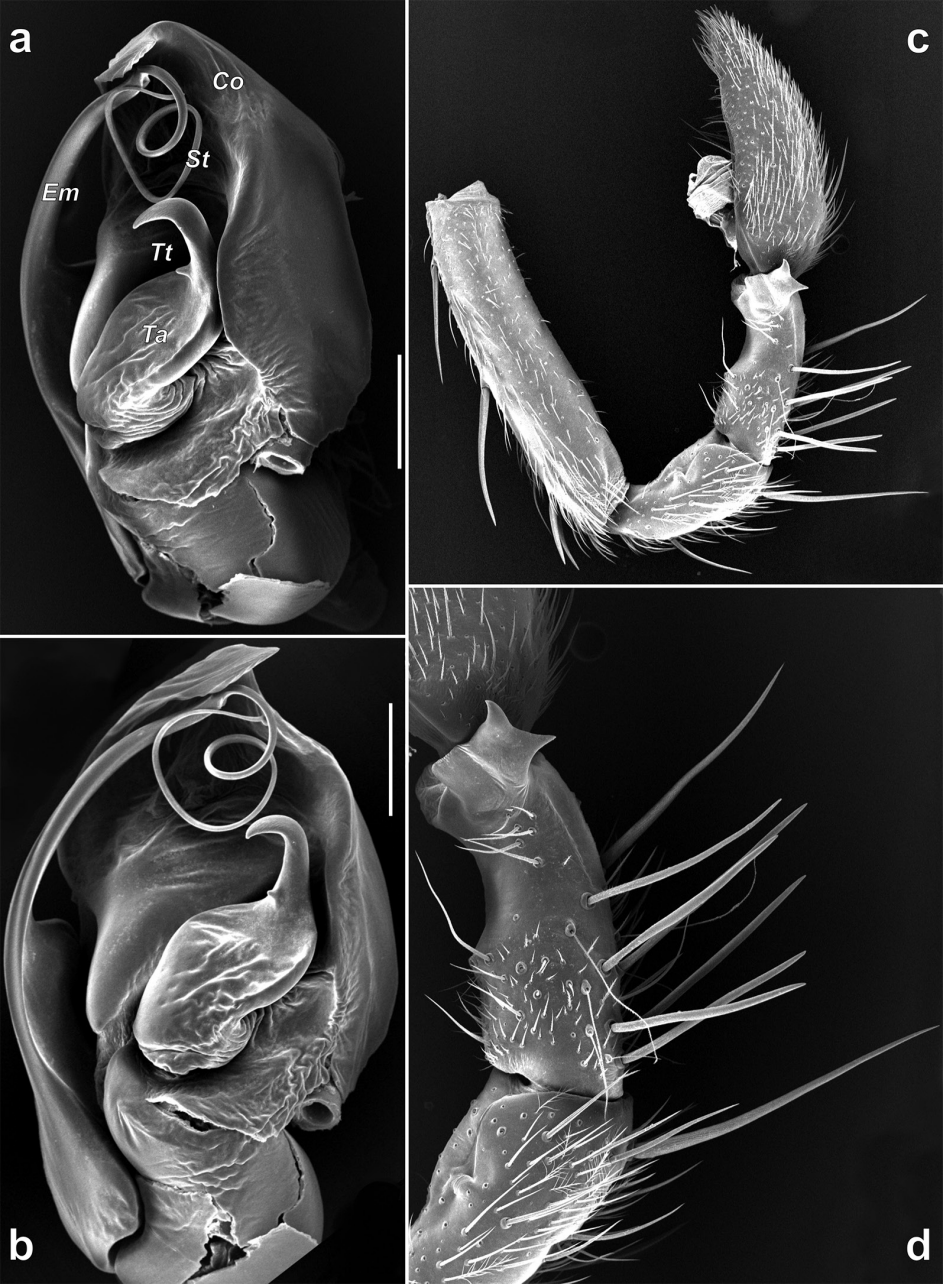
Male palp of *Pterotrichastrandi*. **a–b** bulb, retrolateral and ventral **c** palp, retrolateral **d** palpal patella and tibia, lateral. Scale bars: 0.1 mm. Abbreviations: *Co* conductor; *Em* embolus; *St* stylus; *Ta* tegular apophysis; *Tt* tooth of tegular apophysis.

Figs 6 , 7 , 8 , 9 , 15c , 16

Pterotricha strandi Spassky, 1936: 37, f. 1–3 (♂); Marusik 2016: 279, f. 1–13 (♂).
Bobineus löffleri Roewer, 1955: 774, f. 23a–g (♂). **Syn. n.**
Pterotricha tikaderi Gajbe, 1983: 95, f. 1A–H (♂). **Syn. n.**
Pterotricha loeffleri : Marusik et al. 2013: 349, f. 1–7, 11–16 (♂♀); Zamani 2015: 13; 2016: 225.

##### Type.

Holotype of *Pterotrichatikaderi* (Figs 6c–f): INDIA: ♂ (ZSI), *Gujarat State*: Dwarki Dist. Jamnagar, 16.02.1975 (V.F. Srivastava).

##### Other material examined.

IRAN: 1♂ (ZUCT), *Isfahan Province*: Shahreza County, March 2015 (A. Zamani); 2♂1♀ (ZUCT), *Hormozgan Province*: Hormuz Island, January 2014 (A. Zamani); 1♀ 1 juv. (ZUCT), *Hormozgan Province*: Parsian, January 2016 (A. Zamani); 1♂1♀ (ZUCT), *Kerman Province*: Baft, Jafriz cave, 14.10.2016 (M.J. Malek Hosseini); 1♀ (EMSUM), *Kohgiluyeh & Boyer-Ahmad Province*: Shadegan, 30°56'24"N, 50°91'99"E, April 2017 (A. Hosseinpour); 1♂ (EMSUMS), same locality and collector, May 2017; 1♂ (EMSUMS), *Kohgiluyeh & Boyer-Ahmad Province*: Pasheh Kaan, 30°31'80"N, 50°81'60"E, April 2017 (A. Hosseinpour); TURKMENISTAN: 14♂ (ZMMU), SW Kopetdagh Mts, 12 km W of Kara-Kala, valley of Su River, 38°24'N, 56°07'E, mountain slope, 24.04.1991 (V.V. Dubatolov).

##### Diagnosis.

Males of this species can be diagnosed from congeners by the square tibial apophysis with sharp corners and strongly erect spines on the palpal tibia (Figs 6c–f, 7a, b, 8a–c). Females of *P.strandi* have massive, unknot looped receptacles and long, sticklike glands that differ from most of congeners (Figure 8d, e).

##### Description.

Well described by Marusik et al. (2013) and Marusik (2016). The male of this species has very long and widely spaced anterior lateral spinnerets, 8 × longer than wide, spaced by 2.5 diameters of a single ALS.

##### Comments.

*Pterotrichaloeffleri* was first described in *Bobineus* Roewer, 1955 (Cithaeronidae) based on the holotype male collected in Tehran Province, and later transferred to *Pterotricha* by Platnick (1991). Marusik et al. (2013) studied the type material and one female specimen collected in Bushehr Province and provisionally considered them conspecific due to the similarities in size and eye pattern and the similarities of the epigyne with the closely related *P.strandi*. Considering that the latter species is poorly illustrated and that the type material was not located, the authors mentioned the probability of the synonymy of the two names (Marusik et al. 2013). Because we were able to collect both sexes of this species from the same localities, we can now confirm that the male and female specimens studied by Marusik et al. (2013) are conspecific. As a result of our survey, we found that this species has a rather broad distribution. Despite differences between Iranian and Turkmenian populations, we consider these as merely variations and therefore, consider *P.loeffleri* a junior synonym of *P.strandi*. Although we were unable to borrow the type material for *P.tikaderi* Gajbe, 1983 (India), based on photographs of the palp (Figure 6c–f) and habitus figures provided to us, we conclude that *P.tikaderi* is also a junior synonym of *P.strandi*.

##### Ecology.

This is a nocturnal spider, mostly hiding beneath rocks and inside crevices during the day and hunting at night. According to our observations, this species doesn’t make silken retreats. It is widespread on the Iranian Plateau, occurring in mountainous areas and sand dunes and sometimes near human dwellings, and two specimens were collected in a cave near the entrance. Mature females can probably be found throughout the year, while adult males can mostly be found from mid-autumn to late spring (Zamani 2016).

##### Records in Iran.

Bushehr, Fars, Hormozgan, Kohgiluyeh & Boyer-Ahmad, Tehran. New records: Isfahan and Kerman (Figure 16).

##### Distribution.

Turkmenistan, Iran, and western India.

#### Iranotricha

Taxon classificationAnimaliaAraneaeGnaphosidae

Zamani & Marusik gen. n.

http://zoobank.org/C9C70DA0-DD13-4199-B5D5-A182CC5B225F

##### Type species.

*Iranotrichalutensis* Zamani & Marusik, sp. n.

##### Etymology.

A combination of Iran, the type locality of the species, and “*tricha*”, referring to the similarity with the genus *Pterotricha.* The gender is feminine.

##### Diagnosis.

The genus differs from all Gnaphosinae by lacking a cheliceral keel. It is most similar to *Pterotricha* by having long anterior lateral spinnerets and the embolus similar to that of *P.cambridgei* (O. Pickard-Cambridge, 1872) and *P.levantina* Levy, 1995 by having a modified anterior part with an invagination. *Iranotricha* gen. n. differs from *Pterotricha* by lacking a cheliceral keel, having the two prolateral teeth greatly reduced and separated from each other (vs. well developed and fused at the bases in *Pterotricha*), having modified setae (long terminal setae (*Ts*), long setae (*Ls*), barbed setae (*Bs*), with longer plumage (*Ss*) on chelicera which are lacking in *Pterotricha* (cf. Figs 11a, b, 12a–c and 12d–f), an embolus with a spine (*Es*) (lacking in *Pterotricha*), a small conductor (smaller and thinner than the embolus vs. larger and wider than the embolus) and an elongate tegular apophysis lacking a large base (vs. tegular apophysis with wide base, much wider than tip).

##### Description.

Same as for the species.

##### Comments.

Long spinnerets and the presence of a sclerotized (non-membranous) conductor behind the embolus indicate that new genus is most probably related to *Pterotricha*. *Iranotricha* gen. n. is also similar to *Scotognapha* Dalmas, 1920, a genus restricted to the Canary Islands (WSC 2018). *Scotognapha* has a greatly reduced, vestigial keel (the new genus lacks a keel) but has plumose “hairs” as in *Pterotricha* and *Iranotricha* gen. n.

##### Composition.

Only the type species.

#### Iranotricha lutensis

Taxon classificationAnimaliaAraneaeGnaphosidae

Zamani & Marusik sp. n.

http://zoobank.org/0CA60690-EB14-4484-B19C-A4DD7D102028

**Figure 10.**
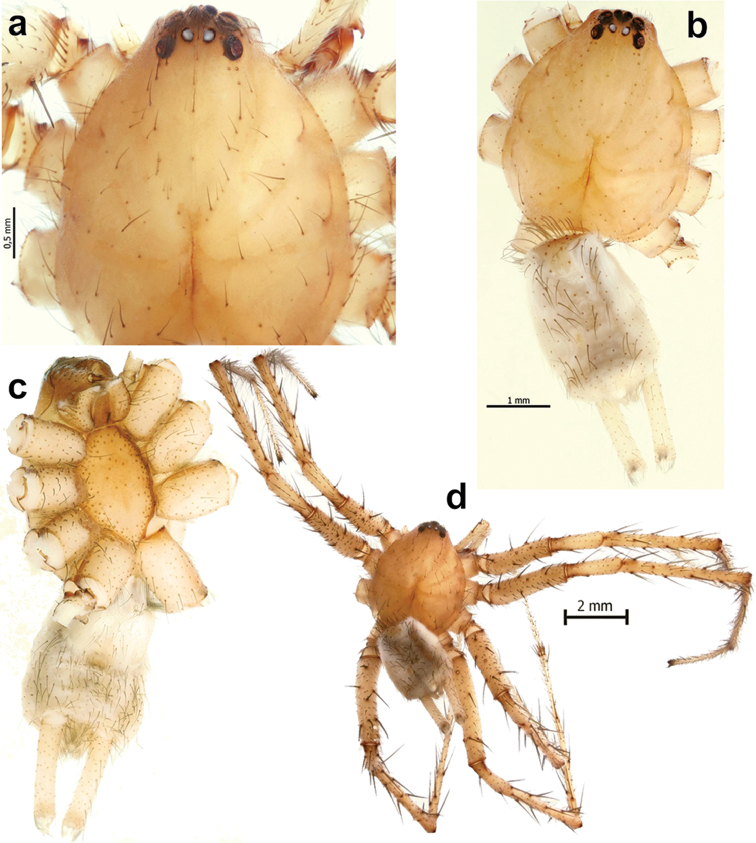
Habitus of *Iranotrichalutensis* Zamani & Marusik sp. n., male. **a** prosoma, dorsal **b–c** body, dorsal and ventral **d** whole specimen, dorsal.

**Figure 11.**
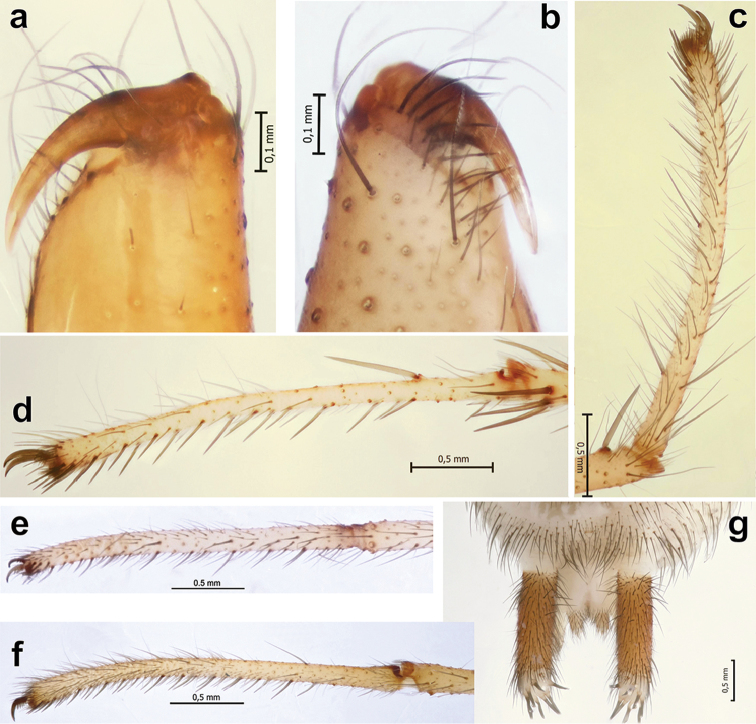
Somatic characters of *Iranotrichalutensis* Zamani & Marusik sp. n. (**a–d**), *Pterotrichastrandi* (**e–f**) and *P.montana* (**g**). **a, b** chelicera, retrolateral and meso-prolateral **c, f** tarsus I, prolateral **d, e** tarsus IV, prolateral **g** spinnerets, ventral.

**Figure 12.**
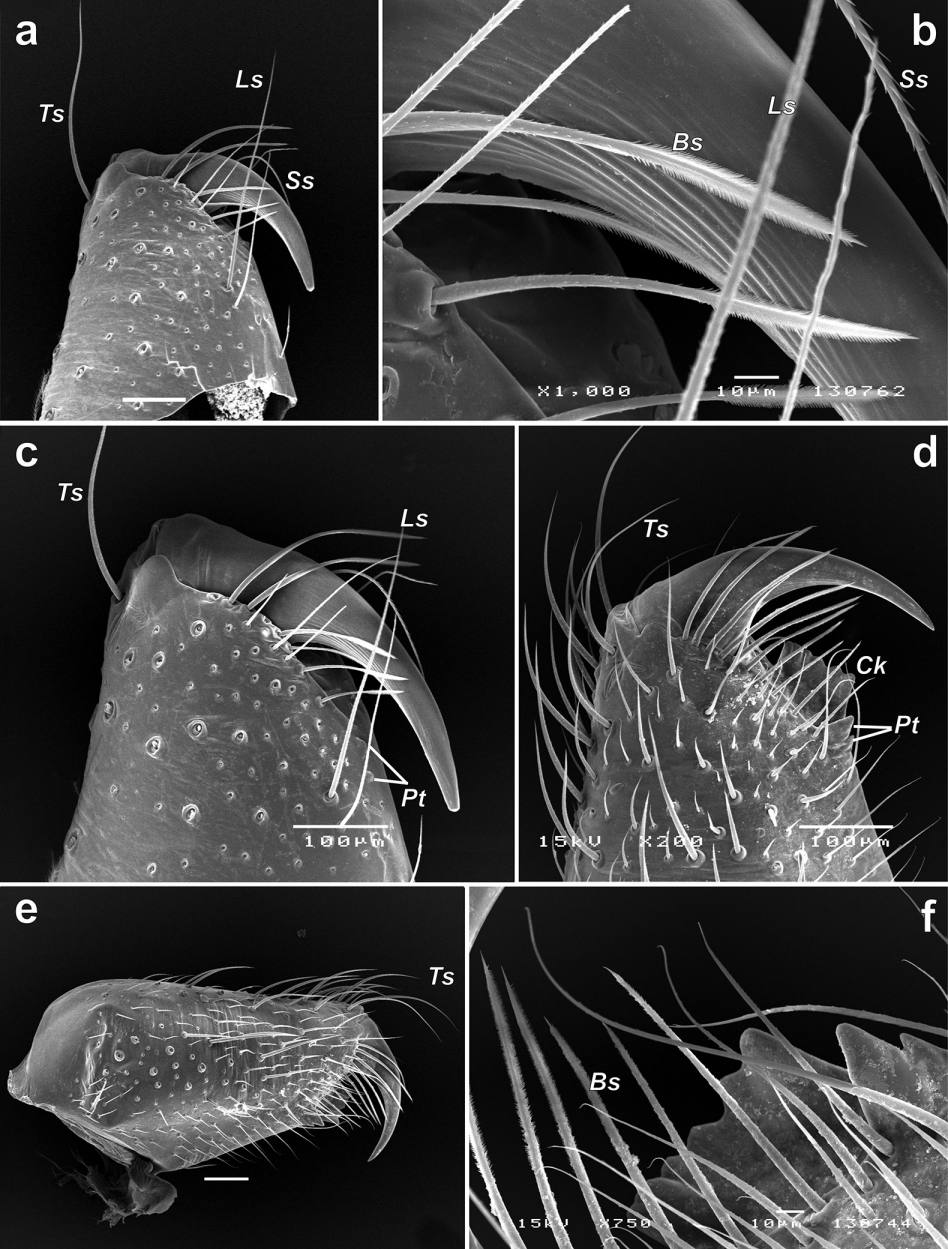
Chelicerae of *Iranotrichalutensis* Zamani & Marusik sp. n. (**a–c**) and *Pterotrichastrandi* (**d–f**). **a, c–d** terminal part, prolateral **b, f** enlarged terminal part of chelicera showing setae and teeth; e whole chelicera, prolateral. Abbreviations: *Bs* barbed seta; *Ck* keel of chelicera; *Ls* long seta; *Pt* prolateral teeth; *Ss* serrated seta; *Ts* terminal seta.

**Figure 13.**
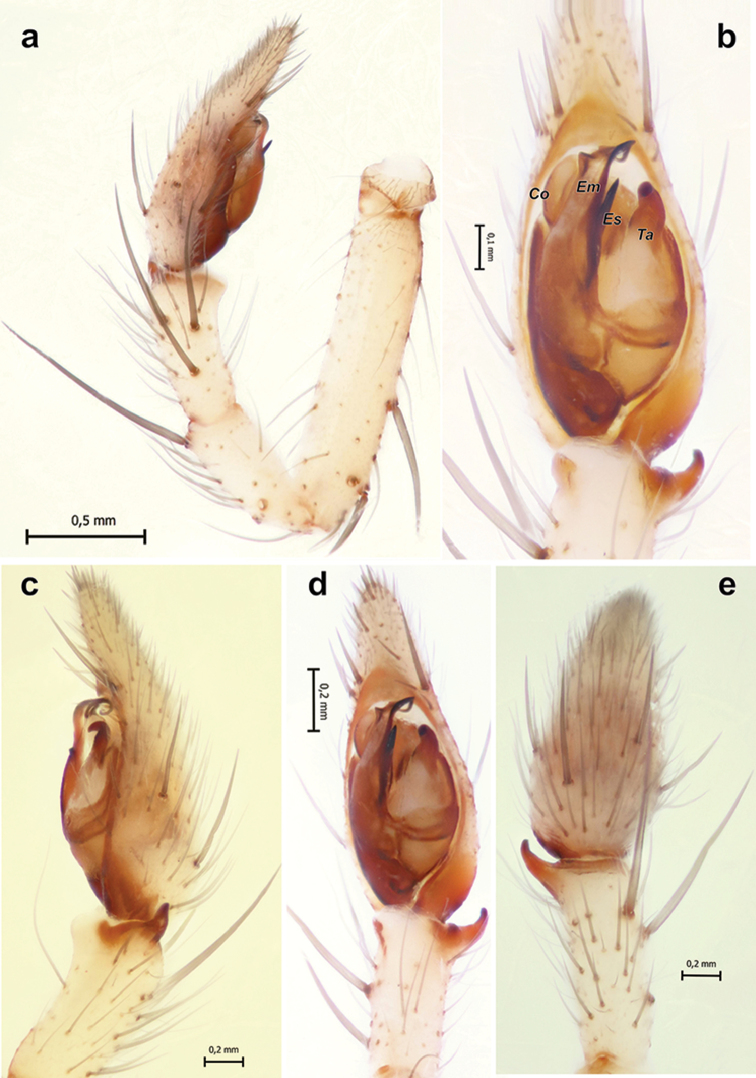
Male palp of *Iranotrichalutensis* Zamani & Marusik sp. n. **a** whole palp, prolateral **b, d** ventral **c** retrolateral **e** dorsal. Spine of the embolus is broken on Figure c. Abbreviations: *Co* conductor; *Em* embolus; *Es* embolic spine; *Ta* tegular apophysis.

**Figure 14.**
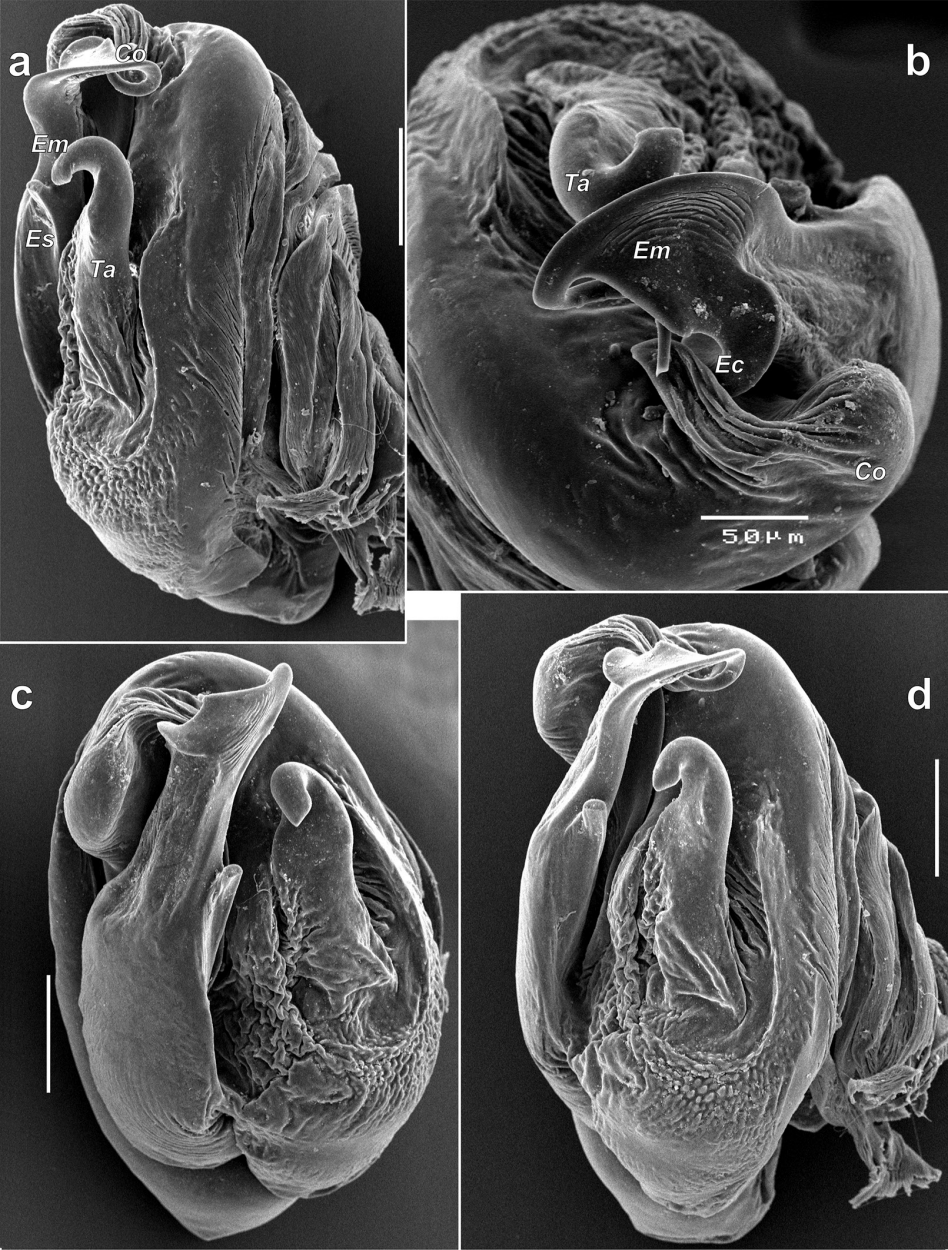
Bulb of *Iranotrichalutensis* Zamani & Marusik, sp. n. **a** retrolateral **b** anterior **c** ventral **d** ventro-retrolateral. Embolic spine is broken. Scale bars 0.1 mm if not otherwise indicated. Abbreviations: *Co* conductor; *Ec* embolic invagination; *Em* embolus; *Es* embolic spine; *Ta* tegular apophysis.

**Figure 15.**
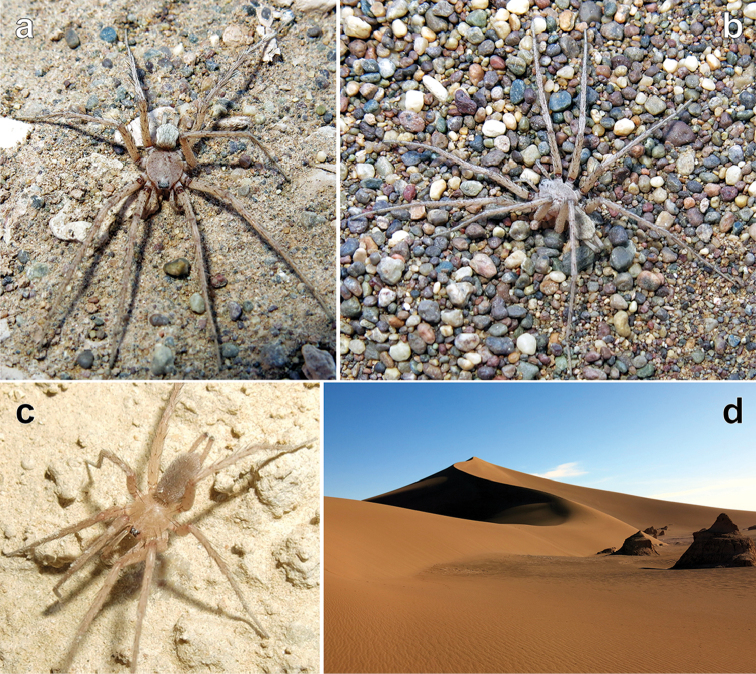
Live male specimens of *Iranotrichalutensis* Zamani & Marusik, sp. n. (**a–b**) and *Pterotrichastrandi* (**c**), and type locality of *I.lutensis* Zamani & Marusik, sp. n. (**d**).

**Figure 16.**
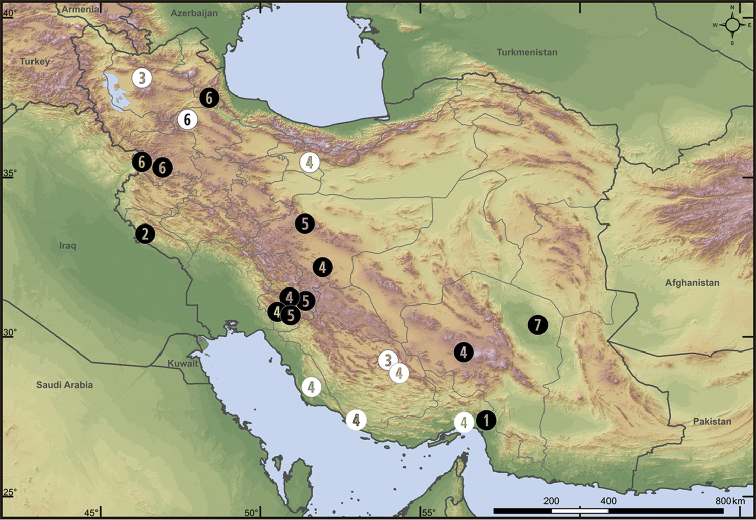
Distribution of *Pterotricha* spp. and *Iranotrichalutensis* Zamani & Marusik, sp. n. in Iran (white symbols refer to literature records, and black symbols refer to our new data): **1**P.cf.dalmasi**2***P.kovblyuki* Zamani & Marusik, sp. n. **3***P.lentiginosa* (?) **4***P.strandi***5***P.montana* Zamani & Marusik, sp. n. **6***P.pseudoparasyriaca***7***I.lutensis* Zamani & Marusik, sp. n.

Figs 10 , 11 , 12 , 13 , 14 , 15a–b, d , 16

##### Type material.

Holotype ♂ (MMUE), IRAN: *Kerman Province*: Lut Desert, Rig-e Setareh, 30°15'26.5"N, 58°42'56.6"E, 252 m, 16.11.2016 (A. Zamani & H. Akhani).

##### Etymology.

The specific epithet refers to the Lut Desert, the type locality of the species.

##### Diagnosis.

The species can be easily recognized from the *Pterotricha* species with long spinnerets by lacking a cheliceral keel. The males of this species can be also recognized due to numerous strong spines on the legs (Figure 10d) and a spine on tarsus IV (Figure 11d). *Pterotricha* species have weak spines and lack a spine on tarsus IV.

##### Description.

Male. Total length 6.45. Carapace 3.7 long, 3.1 wide. Eye sizes and interdistances: AME: 0.14, ALE: 0.21, PME: 0.12, PLE: 0.17, PME-PME: 0.08. Carapace, sternum, labium, chelicerae, and maxillae light brown without any distinct patterns, with scattered short setae and darkening in the ocular area. Chelicera lacking keel but with two strongly reduced (vestigial) teeth that are separated from each other (Figure 12c); a very long terminal seta (*Ts*), 2 long mesal setae (*Ls*), a series of barbed setae (*Bs*) along the prolateral side of the furrow and bent prolateral serrated seta (*Ss*). The barbed setae have long plumage making the wider distally (Figure 12b). Abdomen light grey with long grey setae and a light brown scutum anteriorly. Legs yellow, with numerous spines, including one spine on tarsus IV. Scopula on metatarsi and tarsi indistinct; tarsus I with more thick and thin macrosetae than tarsus IV (Figs 11c–d). Tarsi of all legs with cuticular cracks (pseudosegmented). Leg measurements: I: 15.85 (3.85, 1.9, 3.6, 4.05, 2.45), II: 17.10 (4.0, 2.0, 4.10, 4.30, 2.70), III: 16.41 (3.9, 1.70, 3.95, 4.35, 2.51), IV: 19.1 (4.3, 2.0, 4.1, 6.0, 2.70). Anterior lateral spinnerets almost 6 x longer than wide and almost as long as abdomen width.

Palp as in Figs 13–14; patella and tibia elongate, almost as long as femur and longer than cymbium; patella with very strong and long macrosetae, >1.5 x longer than tibia; tibia cylindrical, unmodified; tibia with relatively small retrolateral apophysis (not longer than diameter of tibia) with tip bent anteriorly, prolateral side with three strong and long macrosetae of equal length to the tibia; cymbium long, approx. 3 x longer than wide with three strong dorsal macrosetae; tegular apophysis elongate, almost cylindrical, with unmodified base; conductor small, partly hidden by embolus; embolus broad at the base, with a strong retrolateral spine (*Es*); anterior part of embolus modified, widened, with an invagination (*Ec*) corresponding (fitting) to conductor (*Co*), tip of embolus stylus-like, looped, directed dorsally and terminating at the tip of the conductor

Female. Unknown.

##### Comments.

It is unclear whether the opening of the embolus is at the tip or before the loop, and as this is the only specimen available at this time, we did not dissect it.

##### Ecology.

The holotype was collected wandering on sand dunes in a habitat lacking any vegetation (Figure 15d). Two subadult specimens were also observed (but not collected) in another locality while they were taking refuge under two large stones. Recently, the hottest place inhabited by spiders was reported to be the Death Valley, Inyo, California, with the highest ground temperature measured at 56.7 °C (Mammola et al. 2017), but the sand surface of the Lut Desert, where the holotype was collected, has been recently measured at temperatures as high as 78.2 °C (Akhani and Aghakouchak pers. comm., Zamani and Marusik 2018).

##### Records in Iran.

Kerman (Figure 16).

##### Distribution.

Lut Desert, southeastern Iran.

## Conclusions

As a result of this study, the number of species of *Pterotricha* known from Iran increased from three (Zamani et al. 2018) to six. Two of the species are currently known only from Iran (*P.kovblyuki* Zamani & Marusik, sp. n., *P.montana* Zamani & Marusik, sp. n.), and one is known only from Iran and adjacent Azerbaijan (*P.pseudoparasyriaca*). As a result of two new synonymies, the range of *P.strandi* is broadened, representing one of the largest ranges in the genus. We assume that the actual number of species in this genus occurring in Iran is higher considering that many remote, desert habitats have not been properly investigated regarding arachnofauna.

## Supplementary Material

XML Treatment for Pterotricha

XML Treatment for Pterotricha cf. dalmasi

XML Treatment for Pterotricha kovblyuki

XML Treatment for Pterotricha lentiginosa

XML Treatment for Pterotricha montana

XML Treatment for Pterotricha pseudoparasyriaca

XML Treatment for Pterotricha strandi

XML Treatment for Iranotricha

XML Treatment for Iranotricha lutensis
